# Inkjet printed IGZO memristors with volatile and non-volatile switching

**DOI:** 10.1038/s41598-024-58228-y

**Published:** 2024-03-29

**Authors:** Miguel Franco, Asal Kiazadeh, Jonas Deuermeier, S. Lanceros-Méndez, Rodrigo Martins, Emanuel Carlos

**Affiliations:** 1 Center of Physics, University of Minho and Laboratory of Physics for Materials and Emergent Technologies, LapMET, Campus de Gualtar, 4710-057 Braga, Portugal; 2 CENIMAT|i3N, Department of Materials Science, School of Science and Technology, NOVA University Lisbon and CEMOP/UNINOVA, Caparica, Portugal; 3 BCMaterials, Basque Center for Materials, Applications and Nanostructures, UPV/EHU Science Park, 48940 Leioa, Spain; 4 IKERBASQUE, Basque Foundation for Science, 48009 Bilbao, Spain

**Keywords:** IGZO, Memristor, Inkjet printing, Volatile and non-volatile behavior, Printed electronics, Electronic devices, Electronic devices

## Abstract

Solution-based memristors deposited by inkjet printing technique have a strong technological potential based on their scalability, low cost, environmentally friendlier processing by being an efficient technique with minimal material waste. Indium-gallium-zinc oxide (IGZO), an oxide semiconductor material, shows promising resistive switching properties. In this work, a printed Ag/IGZO/ITO memristor has been fabricated. The IGZO thickness influences both memory window and switching voltage of the devices. The devices show both volatile counter8wise (c8w) and non-volatile 8wise (8w) switching at low operating voltage. The 8w switching has a SET and RESET voltage lower than 2 V and − 5 V, respectively, a retention up to 10^5^ s and a memory window up to 100, whereas the c8w switching shows volatile characteristics with a low threshold voltage (Vth < − 0.65 V) and a characteristic time (*τ*) of 0.75 ± 0.12 ms when a single pulse of − 0.65 V with width of 0.1 ms is applied. The characteristic time alters depending on the number of pulses. These volatile characteristics allowed them to be tested on different 4-bit pulse sequences, as an initial proof of concept for temporal signal processing applications.

## Introduction

The arrival of the Internet of Things (IoT)^1,2^, and Industry 4.0, both revolutionized the requirements for smart devices, requiring high power efficiency while keeping the manufacturing costs low. To keep the costs down and reduce waste and environmental impact, additive manufacturing technologies have faced increasing adoption to manufacture smart devices^3,4^. Machine learning (ML) is becoming an increasingly relevant technology in many industries that rely on artificial intelligence. To facilitate the widespread integration of ML, it is imperative to enhance computational efficiency through the adoption of innovative computing architectures. This endeavour aims to improve performance, achieve power efficiency, and mitigate carbon emissions, thereby contributing to the establishment of a more sustainable computational paradigm^5^.

Memristors are two-terminal devices with non-linear electrical characteristics which can be broadly classified into volatile and non-volatile, depending on their ability to maintain their current resistive state upon the removal of the electrical bias. Memristors show short and long-term memory^6^, which have significant potential applications across diverse areas. These properties grant an opportunity for expanding the development of neuromorphic computing. In this field, memristors offer the prospect of building circuits that simulate the parallel computation capabilities of the human brain^7^, leading to more efficient artificial intelligence computing. The compact structure allows high density circuits and the ability to both store and process information at the same physical locations, make memristors suitable candidates for neuromorphic computing applications. The device shows the ability of changing its conductance under the application of an electric field. The active layer can be based on inorganic materials such as transition metal oxides^8,9^, organic materials^10^ or a blends of both (e.g. metal-coordinated azo aromatics^11,12^). The change in conductance typically involves the creation and disruption of conductive filaments (CFs)/interface/bulk modulation comprising a localized concentration of defects^13^. Amorphous metal oxide semiconductor (AOS) materials, such as amorphous indium-gallium-zinc oxide (IGZO), have been widely studied and applied in thin-film transistors (TFTs)^14–16^ for display applications as switch and driver of LEDs due to high transparency, carrier mobility, and environmental stability^15,17^. Recently IGZO has started to generate interest as an active layer in memristors^17–20^. IGZO exhibits resistive switching (RS) which strongly depends on oxygen vacancies (Vo) present in IGZO. In solution processes, the Vo can be controlled by changing the gallium concentration. A change in IGZO molar proportion, particularly an increase of gallium will suppress the formation of the oxygen vacancies, resulting in more resistive devices^21^. If the Ga molar ratio is too high the IGZO films are too resistive to display resistive switching properties. Moreover, the IGZO films must have a sufficient concentration of oxygen vacancies to show resistive switching. It has been reported that solution processed IGZO^17^ achieves the optimal resistive switching behaviour at a stoichiometry of IGZO of 1:3:1. A big advantage of using AOS in memristor technology is the integration of the electronic support. Adopting AOS,enables the creation of circuits where the memristors are made with the same materials as active devices such as transistors, thus minimizing manufacturing steps and greatly reducing manufacturing costs^22^.

Responsible electronics can contribute to strongly reducing the environmental impact and material waste of the electronic industry by shifting from traditional manufacturing industrial methods to innovative methods and materials with lower negative environmental impact. The most used method to deposit IGZO thin films is sputtering as leads to a highly uniform deposition in a controlled environment^23^. However, this technique has low deposition efficiency due to the coating of more area than just the substrate (chamber walls) and the subsequent substrative processes to pattern IGZO which leads to high waste of the initial material from the target^24^.

Printed electronics is becoming an alternative to conventional photolithography technology to minimize material waste. It consists of the deposition of an ink on a substrate, that can be paper^25^, textile^26^, glass^27^, and polymer^28,29^, being compatible with the manufacturing of flexible and wearable devices. Printing techniques allow to produce electronic devices characterized by their low cost, easy scalability for industrial production, and flexibility, being also an environmental friendlier technology^30^.

In particular, inkjet printing is a digital technique that allows low-cost deposition of patterned thin films with a controlled thickness. Inkjet printing has remarkable advantages for electronic circuits: it is a digital process, no masks or screens are necessary; it is an additive process, materials are applied only where desired, consequently the waste generated is minimal and it is a non-contact process, the print nozzles do not contact the substrate, preserving delicate surfaces. Compared to conventional microelectronic technologies, the inkjet printing technique achieves a reduction of cost of 64%^31^. The characteristics of inkjet technique allows for the fabrication of highly uniform memristors with low performance variations without using photolithography techniques. Despite the interest, just a few works have been reported on inkjet printed memristors^17,25,27,32–40^.

Memristors can be divided into volatile and non-volatile types. For non-volatile memristors, the state can be maintained after the removal of voltage bias. On the other hand, volatile memristors will spontaneously return to the high resistance state (HRS) in milliseconds to nanosecond after the removal of the external excitation^41^. In a memristor array the non-volatile resistive switching behaviour allows it to perform vector–matrix multiplication, a crucial operation in artificial neural network hardware^42^. Volatile memristors can dynamically adjust their resistance showing short term memory depending on time-varying signal schemes. This could lead to improved performance in tasks that involve temporal dynamics such as reservoir computing (RC). The RC is not explicitly trained for a specific task. Instead, the training focuses on adjusting the readout weights that transform the reservoir's dynamics into the desired output. The readout function in a RC system is typically simple and easy to train, based on a linearly weighted combination of the reservoir neuron node value^43^.

Regarding hardware implementation of RC, memristor crossbars^36,43–47^ or memristive networks relying on random nanoparticles^48^ as reservoir are reported for real-time handwritten digit recognition^43,47,48^, neural activity analysis and speak recognition^44–46^. One of the advantages of RC are the reduction of the size and training cost^43^. One potential representative application of the above systems combined with printed techniques is in the area of Internet of Medical Things (IoMt), such in cardio monitoring, using the data input to detect arrhythmias^43^. Combining the two distinctive switching modes, it opens the possibility of a cell to be programmed to operate as a volatile memory to use in the reservoir or as a non-volatile memory to use in a readout. So far, to build a complete reservoir system purely based on memristors needs two different types of memristors for each layer^48^.

In this work, we first optimize the resistive switching characteristics of an inkjet printed IGZO memristor and later demonstrate its essential characteristics whose electrical characteristics can be further applied in storage application (non-volatile) or in temporal signal processing (volatile).

## Results

### Influence of IGZO thickness on resistive switching characteristics

To transition into a sustainable economy, it is critical to minimize the waste. In this work, to print a 1 mm^2^ square, only 10 µL of IGZO precursor ink were consumed. In this section the focus will be on the optimization of the IGZO printing and the impact on layer thickness and consequently its resistive switching characteristics.

**Figure 1:**
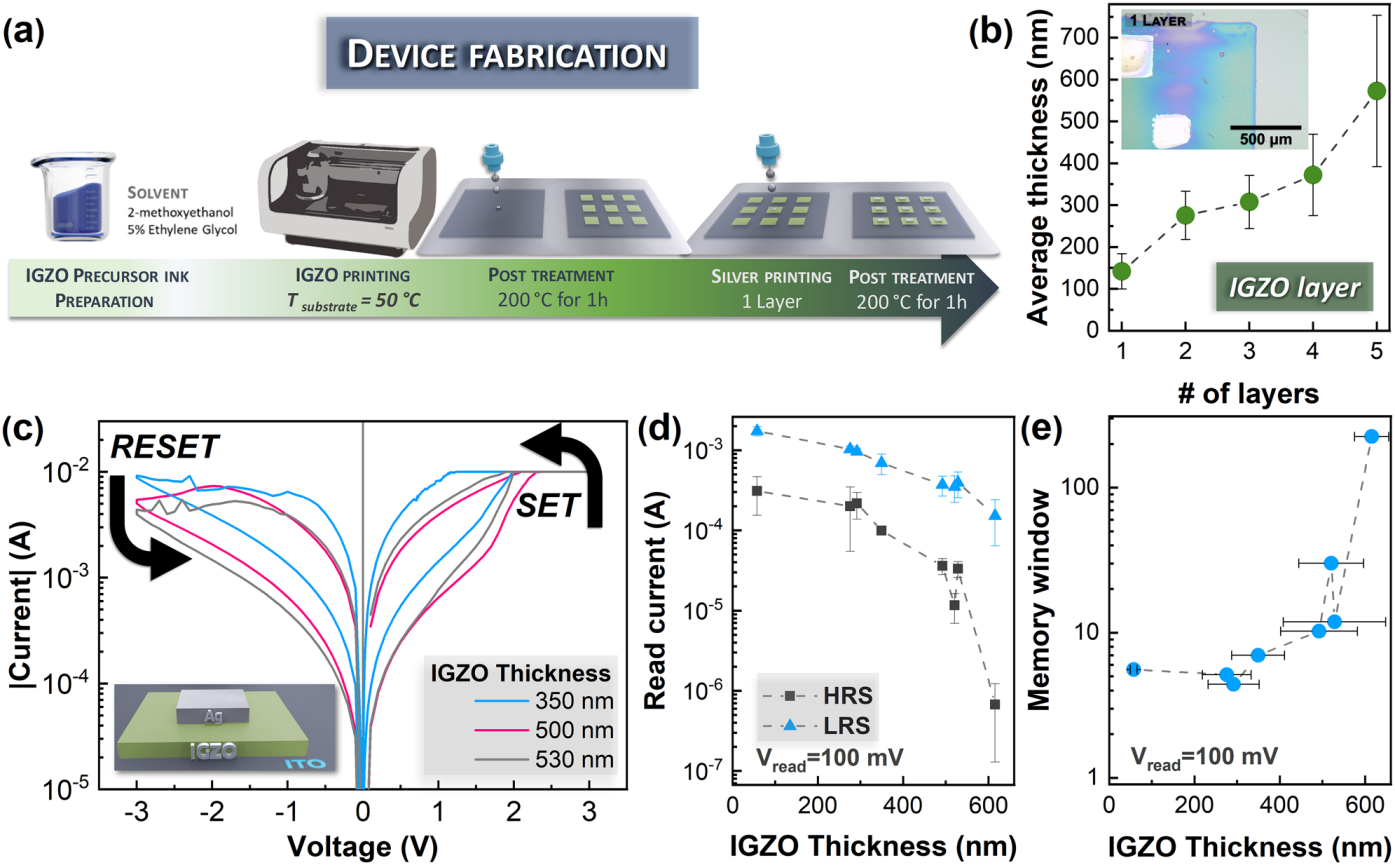
(**a**) Schematic depicting the fabrication of printed Ag/IGZO/ITO memristors on glass substrate by inkjet printing technique (**b**) Average IGZO thickness in function of the number of printed layers with an optical microscope image of the printed Ag/IGZO/ITO memristors as inset. (**c**) I–V characteristics of Ag/IGZO/ITO in function of the IGZO Thickness: 350 nm, 500 nm and 530 nm. (**d**) Read current at 100 mV on HRS and LRS in function of the IGZO thickness. (**e**) respective memory window.

First, to achieve a good quality printed IGZO layer the influence of UV surface treatment before printing each layer was studied. One of the issues in uniformity of inkjet printed films is the coffee ring effect. One approach to mitigate this effect is to heat the substrate during printing^49^, which was adopted in this work. Without any surface treatment, the profilometer data summarized in Fig. 1b and in Figure S1 shows the printed IGZO layer has a low coffee ring and a good coverage without the existence of deep valleys. It is found, that after drying the first IGZO layer, its dimensions shrink 25% to 0.75 × 0.75 mm. Moreover, the IGZO thickness does not scale in a linear trend in function of the number of layers. One printed layer has a thickness of 141 ± 42 µm whereas 5 printed layers have a thickness of 572 ± 181 µm, about 4 times higher than a single layer. The atomic force microscopy (AFM) measurements, shown in Figure S2, are in agreement with the profilometer data. For 1 layer of IGZO, it shows an average thickness of 115 ± 23 nm with an average roughness of 20 ± 5 nm. The average roughness increases to a maximum of 45 nm for 5 printed layers of IGZO. The inset of Fig. 1b shows a microscope image of the printed Ag/IGZO/ITO memristors. The printing definition is great with well-defined borders. On the other hand, when 15 min of UV were applied before printing the IGZO layer, the film shows noticeable overspreading, thus reducing the thickness of the printed IGZO films. The thickness of 1 and 5 layers are 15 ± 10 µm to 319 ± 40 µm, respectively (Figure S3), a respective decrease of 90% and 45% when compared to the non-treated films.

Figure 1c shows the influence of the device characteristics in function of the IGZO thickness. Regardless the thickness, the devices show gradual switching in both SET and RESET. Moreover, the devices became more resistive with the increase of the thickness (Figure S4). For an IGZO thickness between 50 and 620 µm, the average read current drops from 1.7 ± 0.2 mA to 0.15 ± 0.09 mA for LRS and from 0.3 ± 0.1 mA to 0.7 ± 0.5 µA for HRS (Fig. 1d). The HRS current decreases with IGZO thickness increment. Also, the memory window, ratio between the LRS and HRS current, increases in an exponential trend from 5 up to 200 (Fig. 1e,h).

**Figure 2:**
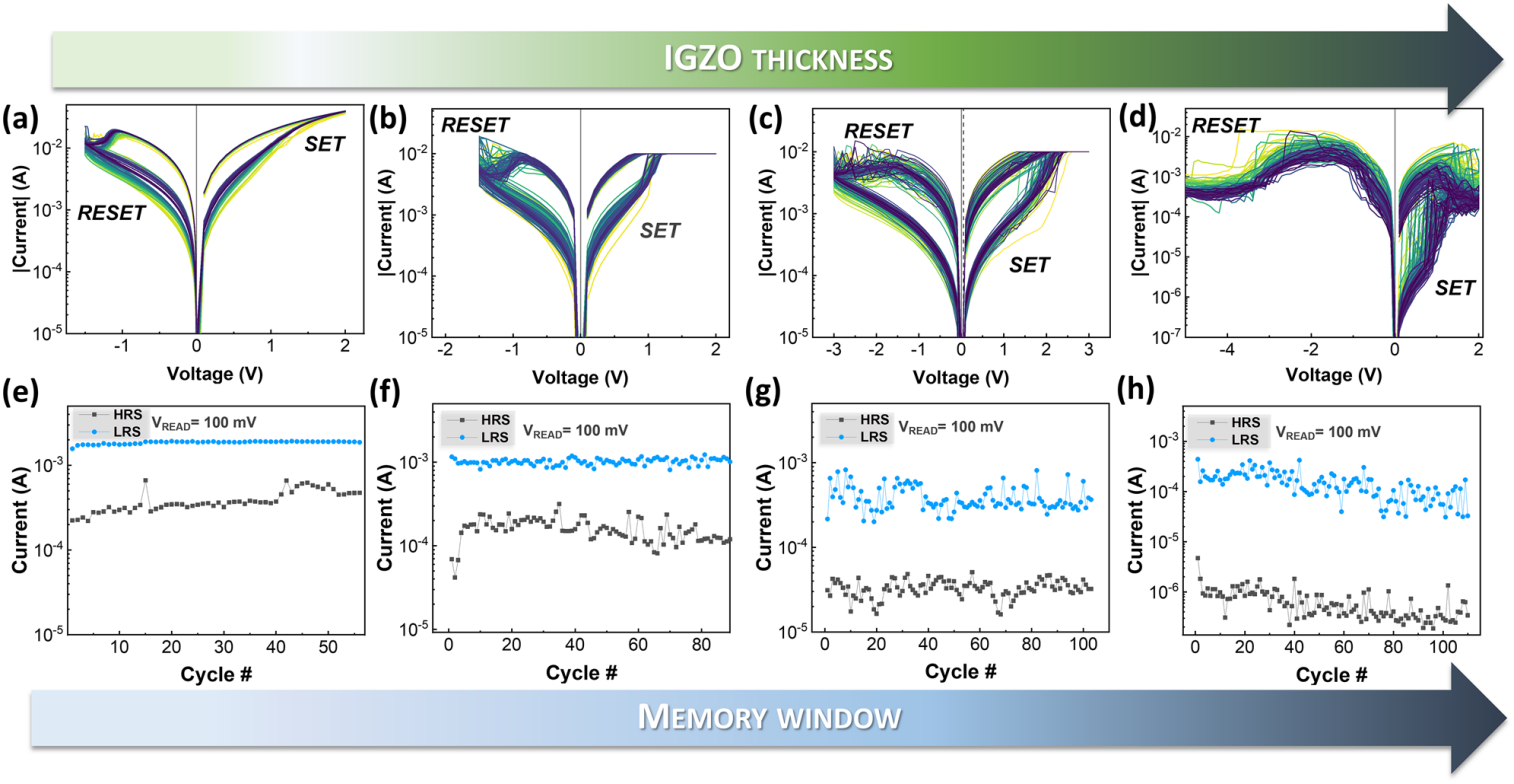
Endurance I–V sweeps (top row) and respective endurance data (bottom row) of Ag/IGZO/ITO memristors in function of IGZO layer thickness: (**a**), (**e**) 50 nm (**b**), (**f**) 275 nm (**c**), (g) 500 nm (**d**), (h) 620 nm.

Figure 2(a,d) shows the endurance curves of Ag/IGZO/ITO memristors with different IGZO thickness with respective current levels when reading at 100 mV Fig. 2(e,h). The devices show stable switching with low dispersion. Both SET and RESET voltages increase with the increase of IGZO thickness, from 1 to 2 V and from − 1.5 V to − 5 V for SET and RESET, respectively. There is a decrease in cycle variation with the decrease of thickness. Moreover, the switching became more gradual with the decrease of thickness. However, the trade-off is the decrease of memory window. Also, the IGZO devices with the lowest thickness (50 nm) show an increase of the HRS current during cycling, thus gradually reducing the memory window during endurance test. For the thicker devices that trend in HRS current does not occur. Moreover, the existence of a secondary volatile switching with opposite polarity was found (Figure S5).

**Figure 3:**
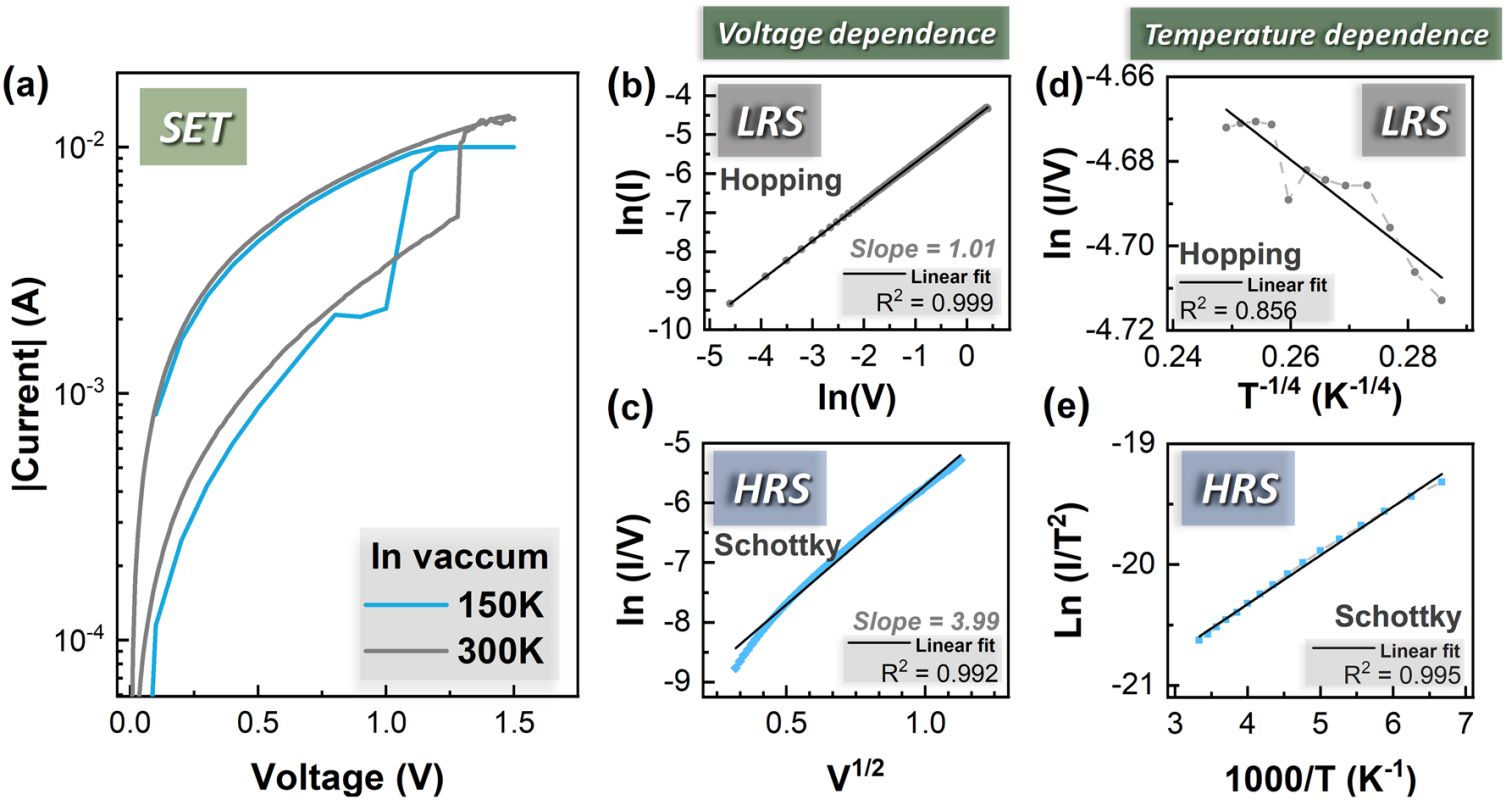
Study of the mechanism of Ag/IGZO/ITO devices. Fitting of the SET curve: (**a**) on LRS for hopping and (**b**) on HRS for Schottky emission. Temperature measurements carried out in vacuum from 150 to 300 K with a step of 10 K on Ag/IGZO/ITO memristors: (**c**) SET sweep at 150 K and 300 K, (**d**) read current on LRS and HRS from 150 to 300 K, (**e**) Fitting mechanism of the LRS for hopping, (**f**) Fitting mechanism of the HRS for Schottky emission.

To study the switching mechanism of the Ag/IGZO/ITO memristors, temperature measurements were performed in vacuum from 150 to 300 K. As shown in Fig. 3a, there are no significant changes in current at LRS at 150 K and 300 K, but HRS displays a more pronounced change with temperature. Figure 3b,d show the best current–voltage and current–temperature fittings for LRS. LRS charge transport is assumed to be controlled by variable range hopping (i.e., temperature dependency of 1/T^4^) which is usually reported for strongly disorder systems^50,51^. It is bulk-limited model indicating that defects in IGZO have important roles. The slope double logarithmic plot is 1.01, implying ohmic characteristics as dominated conduction. Therefore, the origin of defects is most probably related to diffusion of silver ions in filament formation. At HRS, the characteristics show a good fit of Schottky emission (thermionic emission) with a good quality fit when plotting the ln (I/V) in function of V^1/2^ also validated by temperature dependency fitting as shown in Fig. 3e.

### Proof of concept of volatile and non-volatile switching characteristics

From the results of the previous section, IGZO with a thickness of 50 nm was chosen for this proof of concept because it leads to a more gradual switching and a lower variability when cycling the devices. These characteristics are important for neuromorphic computing.

**Figure 4:**
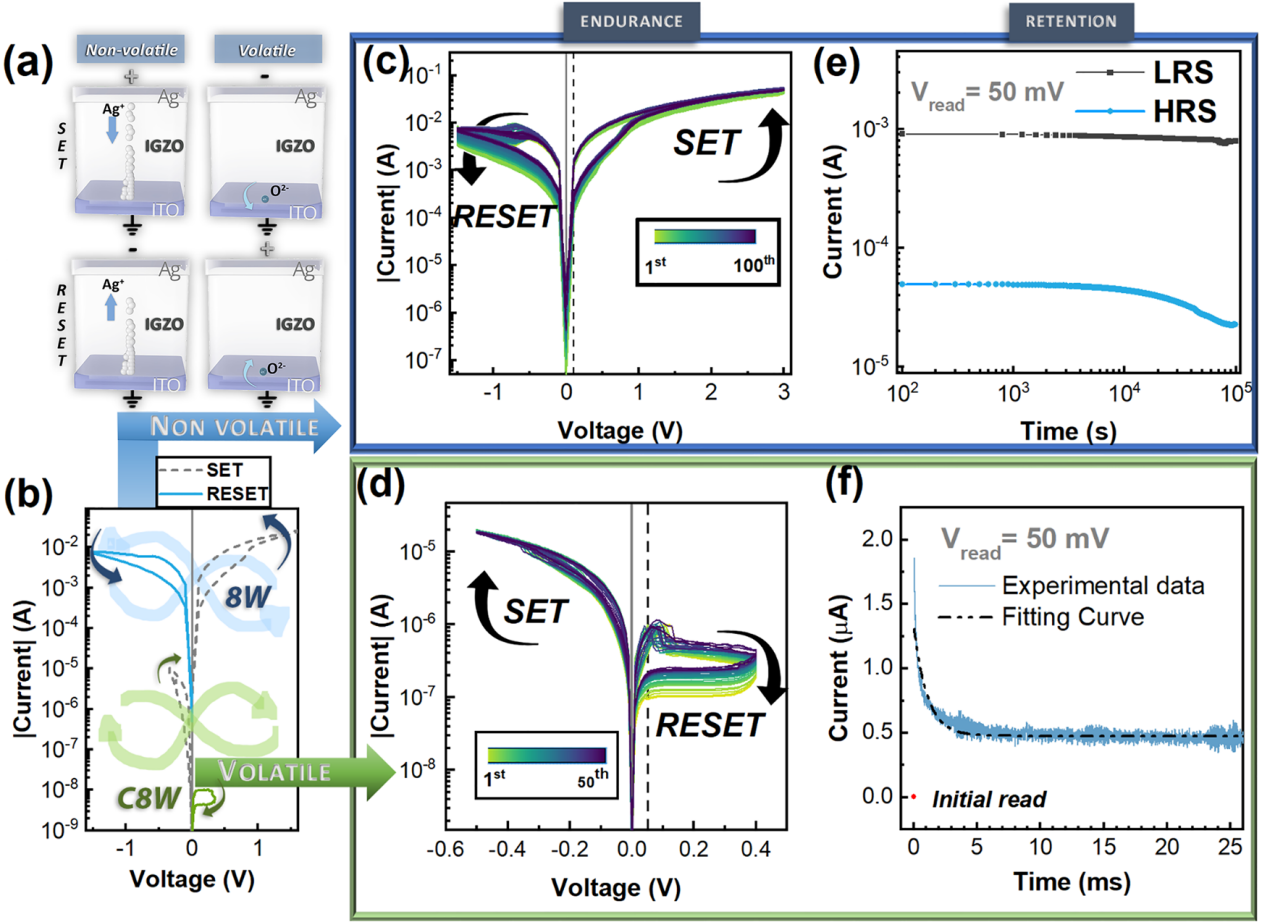
(**a**) Schematic of Ag/IGZO/ITO devices emphasizing the coexistence of two distinctive switching mechanisms. (**b**) Different I–V characteristics of the memristors taken from voltage sweeps: at larger current it follows a counter8wise switching (non-volatile) while for low current it follows an 8wise switching (volatile). (**c**) 100 cycles endurance voltage sweep for non-volatile programming. (**d**) 50 cycles endurance voltage sweep for volatile programming (**e**) Retention test for 105 s at 0.1 V (**f**) Current decay in IGZO memristor after being programmed by 1 write pulse (− 0.65 V, 0.1 ms); the current was then monitored by read pulses at 0.05 V for 25 ms.

The optimized fabricated memristors (the device structure and connection depicted in Fig. 4a, show two distinctive I–V characteristics according to the endurance and current–time relation measurements. The distinct switching mechanisms are explained in the discussion section. There is a significant difference in current levels between two switching modes (Fig. 4b), meaning that only a certain level of current defines resistive switching modes. By standard definition, the electroforming process is the one-time application of a high electric field, higher than the set voltage (V_f_ > V_set_). In the presented devices, in both volatile and non-volatile modes, there is no significant difference between initial SET and other cycles. This way, the device can be categorized as forming-free. In previous works^18,19,52,53^, the IGZO memristors presented a forming-free performance. The devices in their pristine state have a low current (ranging from 10^–10^ A to 10^–5^ A) and a rectification of 1000 (Figure S6).

Using the terminology originated from the works of Dittmann and Waser^54–56^, the switching polarity can be classified into counter-eightwise (c8w) and eightwise (8w) in relation to the active electrode. When the voltage of the active electrode is displayed and the voltage of the other electrode is grounded, then the switching polarity will be called c8w if the SET occurs at negative voltage and reset occurs at positive voltage. The c8w I–V curve (in a linear scale) has a drawing direction which is against that of the handwriting of a (tilted) ‘8’. The opposite switching polarity is called 8w^55^. In this work the voltage is applied to the Ag electrode whereas the ITO electrode is grounded. Since the Ag is a more reactive electrode than ITO, thus we considered the Ag as the active electrode (AE). Therefore, the 8w switching has non-volatile bipolar nature where the c8w switching have volatile characteristics.

In Fig. 4c, the current follows an 8w pinched hysteresis loop with bipolar non-volatile properties (the linear I–V characteristic is shown in Figure S1). The device is compliance free, reaching currents up to 50 mA with a rectification feature. The SET voltage is at 0.8 V while the RESET voltage shows a higher variation being between − 0.5 and − 0.9 V. In the lower voltage regime, however, the direction of SET and RESET are reversed demonstrating so-called c8w switching and volatile resistive switching is obtained. The volatile behaviour shows a very low cycle-to-cycle variability during endurance and a low threshold voltage between − 0.2 V and − 0.3 V (Fig. 4d) with a rectification in the ratio of 100 similar to our previous report^18^ using sputtered IGZO device. Only the c8w switching behaviour shows retention time as shown in Fig. 4e for 10^5^ s.

The short-term memory effect of the memristor for the volatile regime can be described by a time constant τ, from an exponential decay function. Figure 4f depicts the decay curve after a − 0.65 V pulse for 100 µs. The relaxation time, τ, is 0.75 ms. As a result, when programming the device, the device state not only depends on the programming pulse itself, but also on the number of pulses and the pulse intervals.

**Figure 5:**
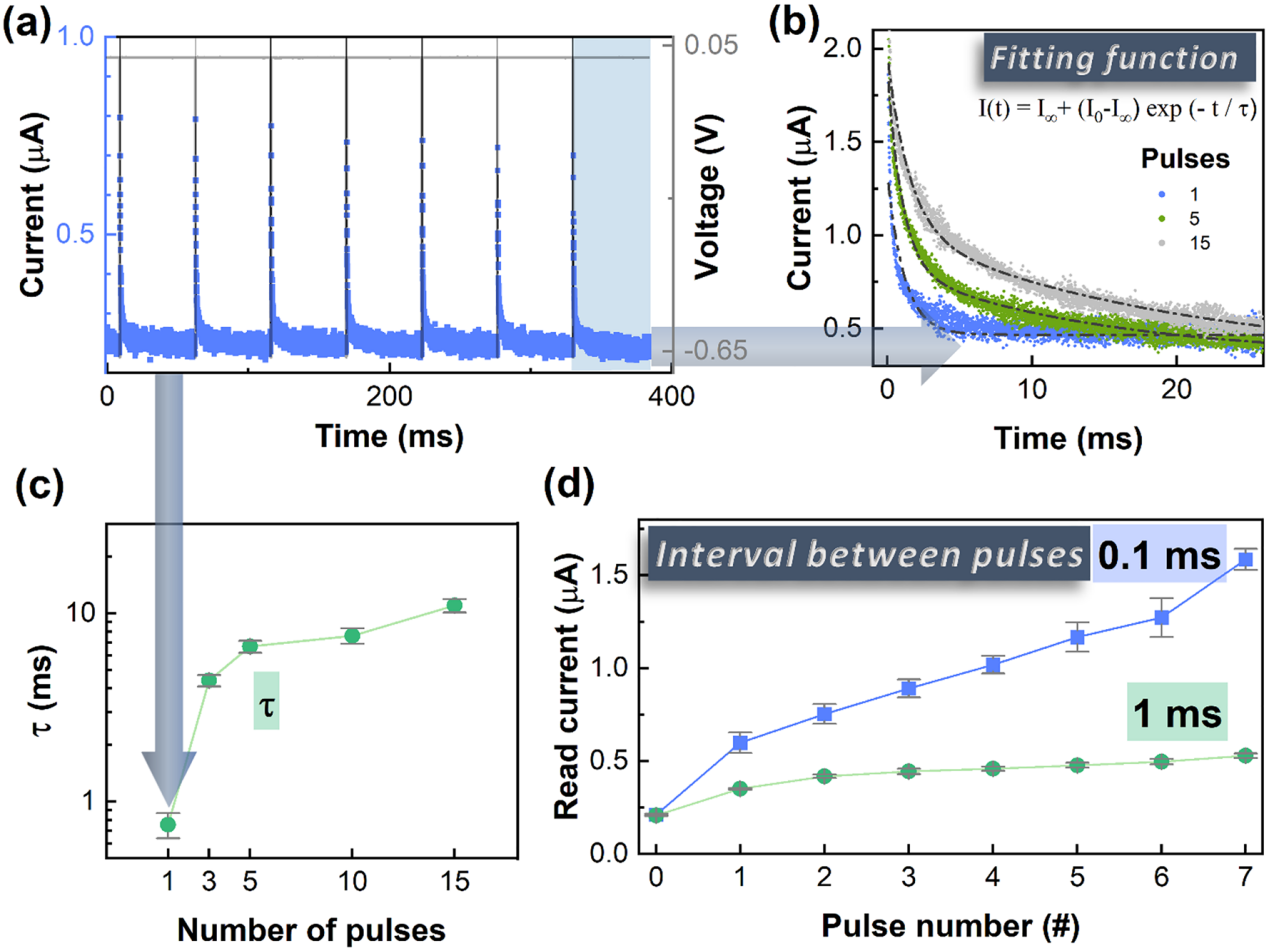
(**a**) Current levels during the application of a − 0.65 V pulse every 60 ms. (**b**) Current decay for 25 ms after being applied different number of pulses (1, 5, 15) with its respecting fitting. (**c**) Time constant (τ) as a function of the number of pulses. (**d**) Read current taken at 0.05 V for 0.1 ms after applying a single pulse with an amplitude of − 0.65 V with different pulse intervals: 0.1 ms and 1 ms.

To demonstrate the similitude amongst the dynamic memory retention of the device and that of the human memory, a single stimulus (− 0.65 V with a duration of 0.1 ms) spaced by a period greatly larger than τ (Fig. 5a) was conducted. The current always stabilizes at 0.3 µA, after the application of a single 0.1 ms pulse. For another test, a 25 ms read pulse at − 0.05 V was applied after different number of pulse stimulations (1, 3, 5, 10, 15). The pulses have an amplitude of − 0.65 V with a duration of 0.1 ms. The results are described in Fig. 5b where a good quality fitting is also presented. Similarly to the results from literature^44^, the current decay follows a simple exponential decay function. Both the relaxation time constant, τ, and the initial current increase with the increasing number of pulses, suggesting that the dynamic retention can be increased by repeating stimulations. The τ increases from 0.75 ms to 11 ms (Fig. 5c) and the initial current increases from 1.8 µA to 2.5 µA.

**Figure 6:**
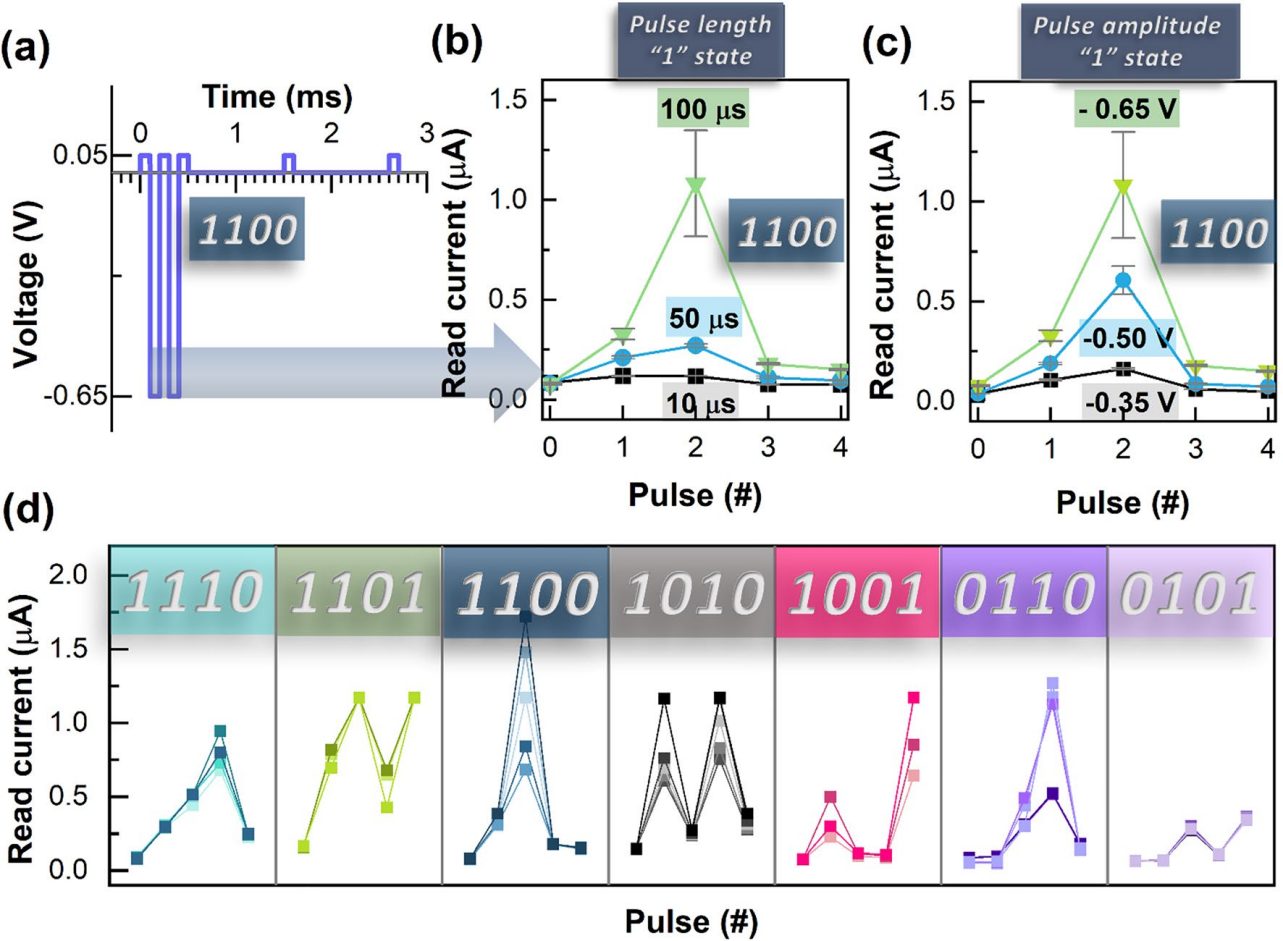
(**a**) 1100 pulse stream with the optimized parameters: for the state 1 it was applied a − 0.65 V pulse for 0.1 ms; the state 0 is 1 ms after the last “1” pulse; the reading was performed at 0.05 V for 1 ms. (**b**) Influence of the pulse length for the “1” state (0.1 ms, 0.5 ms and 0.01 ms) using a [1100] pulse stream. (**c**) Influence of the pulse amplitude for the “1” state (− 0.35 V, − 0.5 V and − 0.65 V) using a [1100] pulse stream (**d**) Read current for various pulse streams using the “1” and “0” pulse parameters in a).

Figure 5d shows the effect of the interval between pulses. After the stimulation, the higher is the interval between pulses, the lower is the read current. For a pulse interval of 0.1 ms, the current reaches 1.5 µA after 7 pulses, whereas for a pulse interval of 1 ms, the current only reaches 0.5 µA. Since the application for temporal signal processing requires very short-term memory, volatile memristors like the one presented in this work are a great potential candidate. Figure 6a shows the volatile mode memristor device characteristics to different temporal inputs.

The “1” state corresponds to a pulse with − 0.65 V of amplitude and a width of 100 µs. The “0” state corresponds to the absence of pulses, 0 V amplitude for 1 s. There are 5 read pulses: one when the sequence initiates and then after finishing each state. Figure 6b shows that a pulse width variation of 10 µs, 50 µs and 100 µs can be used to activate the device. The longer the pulse, the higher is the corresponding read current. Also, when applying different pulse voltages (− 0.35 V, − 0.5 V, − 0.65 V), the read current is higher (Fig. 6c).

When a pulse is applied, the state of the memristor will be changed by increasing its conductance and if the pulse interval is short enough, its conductance will be increased. For long intervals the conductance decays to its resting state^43^. Therefore, different temporal inputs will lead to different states of the device.

Figure 6d shows 5 repetitions for the [1110], [1101], [1100], [1010], [1001], [0110] and [0101] sequences, using the same ”0″ and “1″ pulse parameters depicted in Fig. 6a. There is a low variation of the read current over the cycles, however, the trend is the desired one.

The working devices are very consistent in terms of voltage operation (Figure S8a), the same pulse scheme works for the different devices as can be shown in Figure S8b. However, they present some variability regarding the current state due to the presence of pinholes. We also note that reducing the size of the active region can lead to faster switching times, therefore shorter and more intense pulses may induce faster switching.

## Discussion

The devices show a co-existence of threshold switching volatile memristor and bipolar non-volatile switching. The devices can alter from volatile mode (c8w) to non-volatile (8w), but not vice versa. The protocol to change from volatile to non-volatile is by increasing the switching voltage without needing electroforming. The switching polarity is related to the dominated means of the defect redistribution. In VCM filamentary systems, the c8w switching mode the device is SET to its low resistive state (LRS) by applying a negative voltage at the AE and RESET to its high resistive state (HRS) by applying a positive voltage at the AE. The second resistive switching, 8w, occurs at the opposite polarity of the c8w mode. In 8w switching a positive voltage is applied to the AE of the device to bring it to its LRS and a negative voltage is needed to RESET the device to its HRS^57^. On oxide thin film memristors, it has been demonstrated that both switching modes can appear in the same device by changing the operating conditions^54,56,58–61^. In the context of non-volatile 8w switching, the temperature and voltage characteristics suggests that the charge transport in the low-resistance state (LRS) is governed by the variable range hopping model, which is bulk-limited. The electrons are injected into the IGZO without significant potential barrier, and the transport-limiting element is the conduction from defect to defect. Non-volatile switching with silver as active electrode is usually due to metallic cation migration, recognized as electrochemical metallization mechanism (ECM)^62^. This is illustrated in the schematic shown in Fig. 4a. The explanation for the increase in current with increasing temperatures is the nanostructured morphology of the filaments^63^.

The rectification characteristics on the I–V curves on both switching polarities, are due to the presence of small Schottky-type barriers at the interface of Ag/IGZO layers and ITO/IGZO layers for non-volatile 8w switching, and volatile c8w switching respectively. On non-volatile switching at HRS, the characteristics shown a good fit of Schottky emission (thermionic emission). This means that the conduction in the non-volatile HRS is supported by the conduction band of the IGZO and the transport-limiting element is the injection of electrons at the contact interface.

The coexistence of c8w and 8w switching was reported in^58^ for Pt/TiO_2_/Ti/Pt devices where both switching modes occur from the competition between drift/diffusion of oxygen vacancies in the oxide layer and an oxygen exchange reaction across the Pt/TiO_2_ interface. A similar concept can be applied here for the c8w resistive switching, categorized as diffusive memristor following ion exchange at the interface of IGZO and ITO. We have already shown in our previous works^18,19,52,53^, that amorphous oxide semiconductors (AOS)—based memristor present a forming-free performance. One of the main reasons relies on defect profiles of AOS active material at the interface with the electrode which can be easily tuned into distinct resistance states especially in c8w resistive switching behaviour as shown in one or our previous works^64^. The corresponding resistive switching is area-dependent; however, multi- filamentary nature is not excluded.

Moreover, the coexistence of secondary switching in a single memristor cell is usually volatile as reported in^53,56,58^, ^65,66^. In these works, the volatile switching mode is explained by an oxygen exchange reaction between the Pt electrode at the interface with active layer, e.g. metal-oxide.The occurrence of volatile mode at negative voltage polarity laine may be related to ion-related migration at the interface^41^. The exchange of oxygen between ITO and the switching layer can influence the conductivity of the latter^67^. In case of an n-type material like IGZO, the conductivity will increase with the decrease of oxygen content. Hence, under positive polarity at the top electrode, oxygen moves into the ITO layer and gets accommodated as interstitial oxygen, which corresponds to the SET operation. This interstitial oxygen is released back into the IGZO under positive bias during the RESET operation (see Fig. 4a).

## Conclusions

It is demonstrated that solution-based memristors fabricated by inkjet technique have a strong potential for applications due to their scalable production at low cost and low waste formation. In this work, a printed IGZO memristor has been fabricated where only 10 µL of IGZO precursor ink was spent to print a 1 mm^2^ square with minimum waste. The devices shows both volatile and non-volatile behaviour depending on the programming schemes. The IGZO thickness influences the switching voltage and memory window. The non-volatile response follows an 8w switching polarity with a SET and RESET voltage higher than 2 V and − 5 V, respectively, with low cycle variability and a retention up to 10^5^ s and a memory window up to 100. The LRS charge transport is found to be controlled by variable range hopping where the origin of defects on IGZO is most probably related to the diffusion of silver ions in the form of filaments. On the other hand, the volatile switching mode follows an 8w scheme with very low threshold voltage (V_th_ < − 0.65 V) and switching times below 1 ms. The volatile characteristics provide short term retention with a τ of 0.75 ms. Those combined characteristics show that a low-cost technology like printed metal oxide memristors can be used for simple and efficient designs of fully memristive architecture based on IGZO, where the reservoir state (volatile mode) can be processed with the IGZO memristive readout neural network (non-volatile mode). A further step for the demonstration of the system should involve a crossbar design and the corresponding test. Further, it is worth noticing that IGZO memristors can be applied on flexible biocompatible substrates, such as polyimide with parylene as biofriendly encapsulation to be implemented in IoMT device application.

## Methods

### IGZO Precursor ink synthesis and characterization

The IGZO precursor ink synthesis was adapted from^17^ for inkjet printing. Indium(III) nitrate hydrate (In(NO_3_)_3_·*x*H_2_O, Sigma-Aldrich, 99.9%), gallium(III) nitrate hydrate (Ga(NO_3_)_3_·*x*H_2_O, Sigma-Aldrich, 99.9%) and zinc nitrate hexahydrate (Zn(NO_3_)_2_·6H2O, Sigma-Aldrich, 98%) were separately dissolved in a mix of 2-methoxyethanol (2-ME, C_3_H_8_O_2_, Fisher Chemical, 99%) and ethylene glycol (C_2_H_6_O_2_, Sigma Aldrich, anhydrous, 99.8%) in a proportion of (95:5). After a complete dissolution of each precursor, the fuel (urea, Sigma, 99%) was added to each solution and maintained under constant stirring for 1 h. To ensure the redox stoichiometry of the reactions, the urea to In(NO_3_)_3_, Ga(NO_3_)_3_ and Zn(NO_3_)_2_ molar proportions were (5/2):1, (5/2):1 and (5/3):1, respectively. IGZO precursor solution was prepared by combining the three precursor solutions made, to obtain an IGZO molar ratio of 1: 3: 1 for a concentration of 0.2 M. The precursor solution was stirred for at least 36 h at room temperature and filtrated using a PTFE filter (0.45 μm).

The IGZO precursor ink was optimized for inkjet printing while considering the Reynolds (Re), Webber (We) and Ohnesorge (Oh) numbers. The Ohnesorge number is a dimensionless value that describes the tendency for a drop to either stay together or fly apart, by comparing viscous forces with inertial and surface tension forces. The Ohnesorge number is related to the Reynolds number, and Weber number. The value of Z is defined as the inverse of Oh and used to evaluate the drop formation. For a stable drop formation, the value of Z must be between 1 and 10^68,69^. The IGZO ink has a viscosity of 4.16 cP at 20 °C (same temperature condition during printing) and a Z number of 4.8 (Table S1). The Figure S9 shows the Re, We and Oh values for the ink are inside the optimal area for a stable drop formation. The ink viscosity was measured using a Brookfield DV2T viscometer with a speed ranging from 1 to 50 rpm.

### Device fabrication

The developed ITO/IGZO/Ag devices have a common bottom electrode structure. Figure 1a explains the fabrication of the IGZO memristors. The bottom electrode consists of ITO covered commercial glasses. The printing of the IGZO layer was carried out in a Dimatix DMP 2850 inkjet system using a piezoelectric multi-nozzle printing head from Dimatix (DMCLCP-16110) with 10 pL cartridge. The cartridge and stage temperature were kept at 25 °C and 50 °C, respectively. The frequency was 5 kHz and the drop spacing was set to 30 µm. The IGZO layer were printed with an area of 1000 × 1000 µm^2^ followed by a post treatment at 200 °C for 1 h. Before the deposition of the top contacts, a 15 min UV/ozone surface activation was carried. As top contact, silver electrode was used due to facility of deposition with printing techniques^70,71^. Silver inks are very reliable and have a sintering temperature of 200 °C or lower, unlike other metal-based inks which need higher sintering temperatures to be conductive. Therefore, the silver nanoparticle colloidal ink (Sicryst I50T-13 from PV Nano Cell company)) was printed as two subsequent layers with an area of 250 × 250 µm^2^, on top of the IGZO layer by inkjet printing.

The device thickness was measured using a stylus XP-Plus 200 Stylus profilometer from Ambios. The surface morphology of the samples was also determined by Atomic Force Microscopy (AFM), with an Asylum MFP3D. The quasi-static current–voltage (I–V) characteristics and the pulse studies were measured using a Keithley 4200 SCS semiconductor analyser connected to the Janis ST-500 probe station. The signal was applied to the top electrode (Ag) while maintaining the bottom electrode (ITO) grounded. The speed of the measurements was at normal mode with a measurement rate of 50 mV/s without any delay time and the integration time was in auto setting.

### Supplementary Information

Supplementary Information.

## Data Availability

The datasets used and/or analysed during the current study available from the corresponding author on reasonable request.
