# Supplementary material for: Inkjet printed IGZO memristors with volatile and non-volatile switching
Source: Sci Rep. 2024 Mar 29;14:7469. doi: 10.1038/s41598-024-58228-y (PMC10980760; doi:10.1038/s41598-024-58228-y)
Supplement: Supplementary file 1 — Supplementary Information. [file 41598_2024_58228_MOESM1_ESM.docx]

Electronic Supplementary Information (ESI)

**Inkjet printed IGZO memristors with volatile and non-volatile switching**

Miguel Franco^a,b^, Asal Kiazadeh^b*^, Jonas Deuermeier^b^, S. Lanceros-Méndez^a,c, d^, Rodrigo Martins^b^, Emanuel Carlos^b*^

^a^Center of Physics, University of Minho and Laboratory of Physics for Materials and Emergent Technologies, LapMET, Campus de Gualtar, 4710-057 Braga, Portugal

^b^ CENIMAT|i3N, Department of Materials Science, School of Science and Technology, NOVA University Lisbon and CEMOP/UNINOVA, Caparica, Portugal

^c^BCMaterials, Basque Center for Materials, Applications and Nanostructures, UPV/EHU Science Park, 48940 Leioa, Spain

^d^IKERBASQUE, Basque Foundation for Science, 48009 Bilbao, Spain

*Corresponding authors

E-mail:a.kiazadeh@fct.unl.pt, [e.carlos@fct.unl.pt](mailto:e.carlos@fct.unl.pt)

The supplementary information contains relevant data related to the material and electrical characterization of the memristors.

Figure S1 shows the profilometer curve for 1 and 3 layers of IGZO. Figure S2 shows an AFM topography map of IGZO layer with one printing step Figure S3 shows the influence of surface pretreatment on IGZO thickness. Figure S4 shows resistivity of the HRS and LRS in function of device thickness. Figure S5 shows the existence of a secondary volatile switching. Figure S6 shows the pristine state of IGZO memristors. Figure S7 shows the linear characteristics of non-volatile IGZO memristors. Figure S8 shows device variation for a sample of 4 devices. Table S1 and Figure S9 shows the rheological characterization done to the IGZO ink.

**
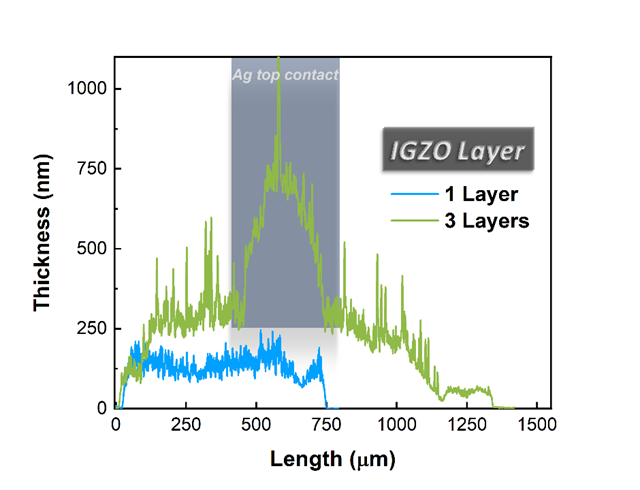
**

**Figure S1** – Profilometer measurement of 1 and 3 layers of IGZO without UV surface pretreatment before printing.


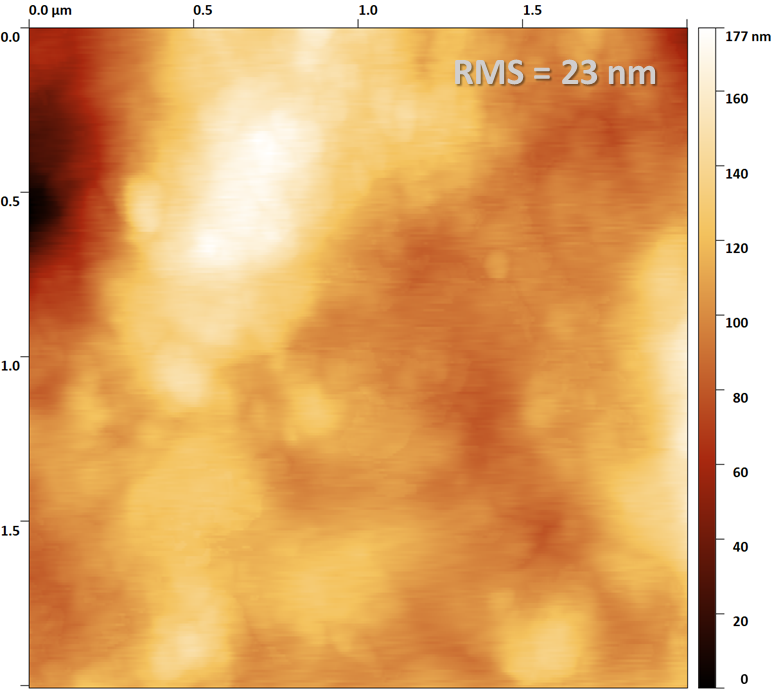


**Figure S2** - AFM topography map of IGZO layer with one printing step and without UV surface pretreatment before printing.


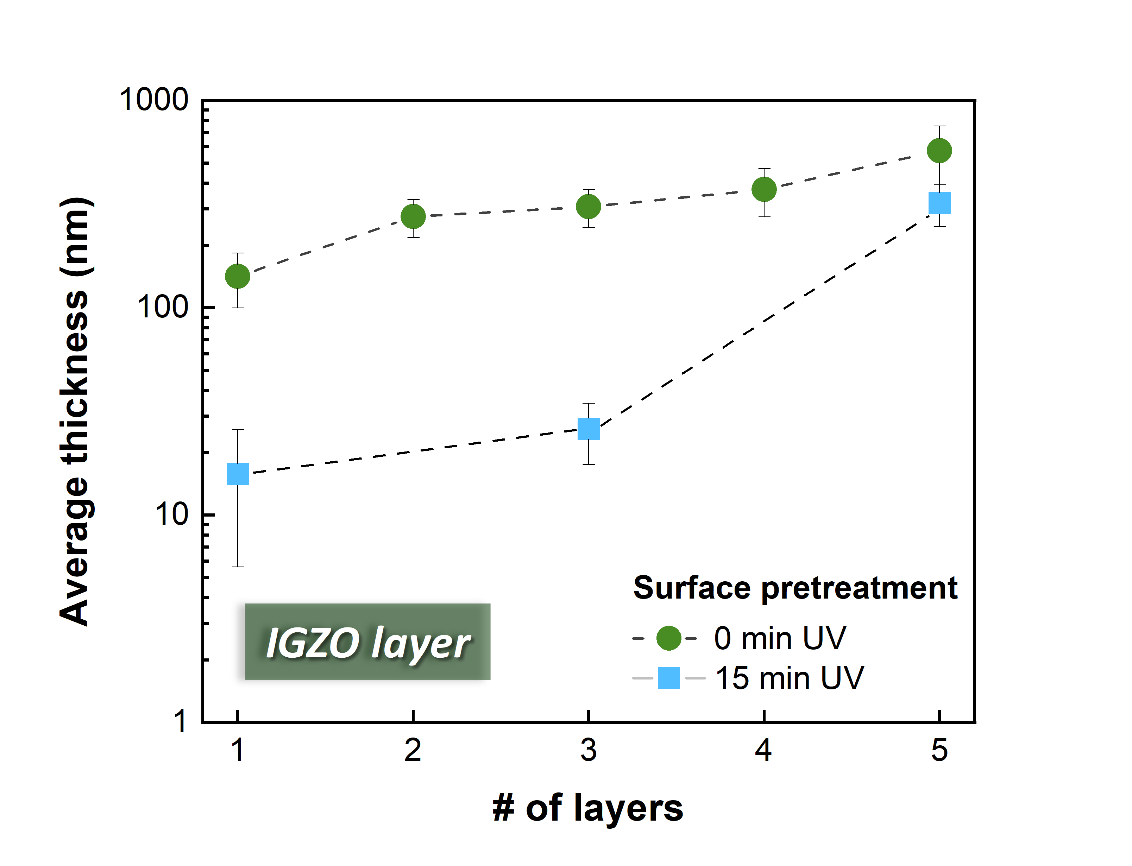


**Figure S3** – Influence of surface pretreatment on IGZO thickness: average IGZO thickness in function of the number of printed layers for no UV surface pretreatment and for 15 min UV pretreatment.


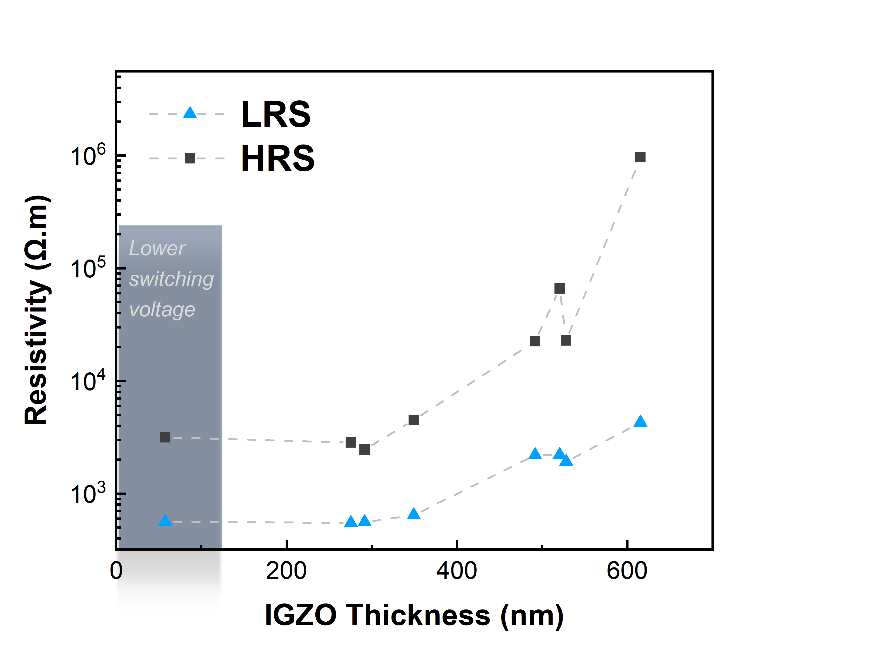


**Figure** **S4** - Resistivity of the HRS and LRS in function of device thickness.


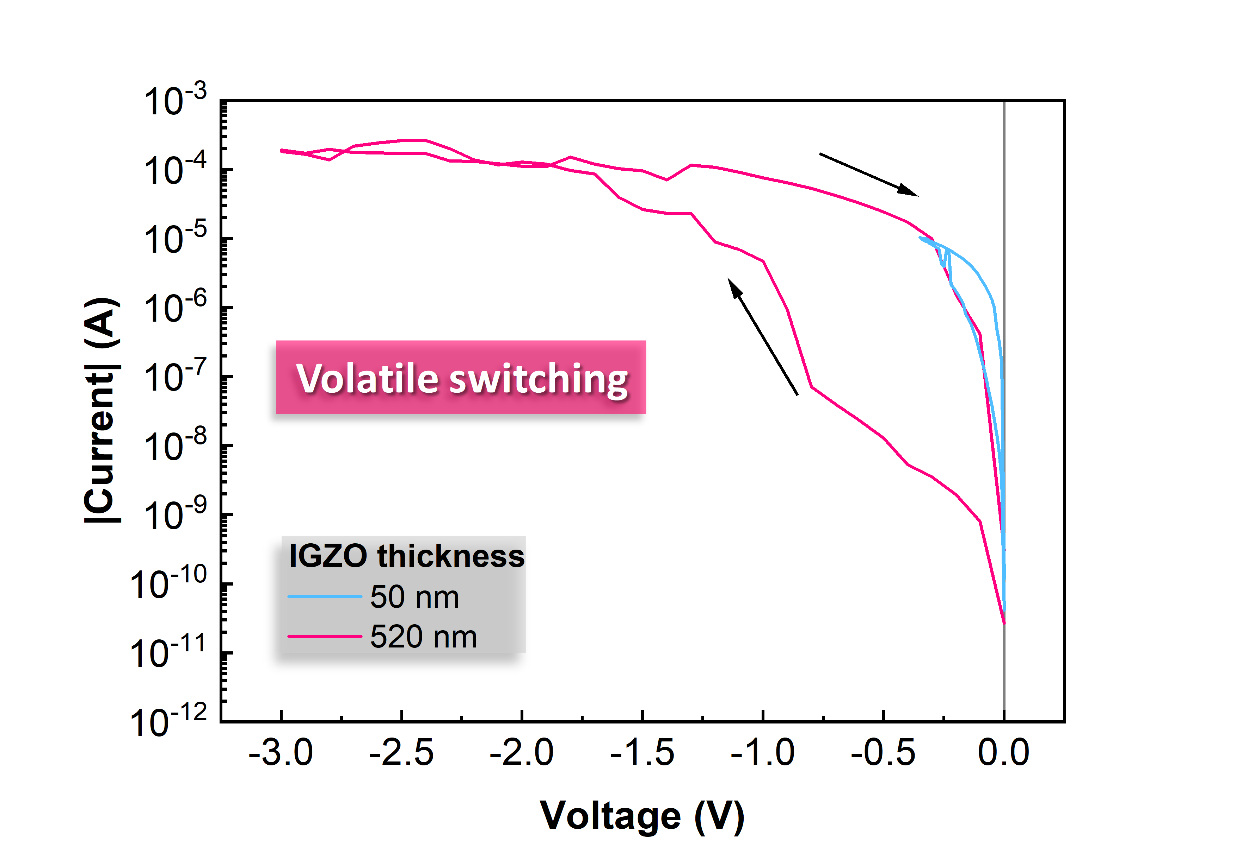


**Figure S5** – Existence of a secondary volatile switching regardless of the thickness of IGZO layer.


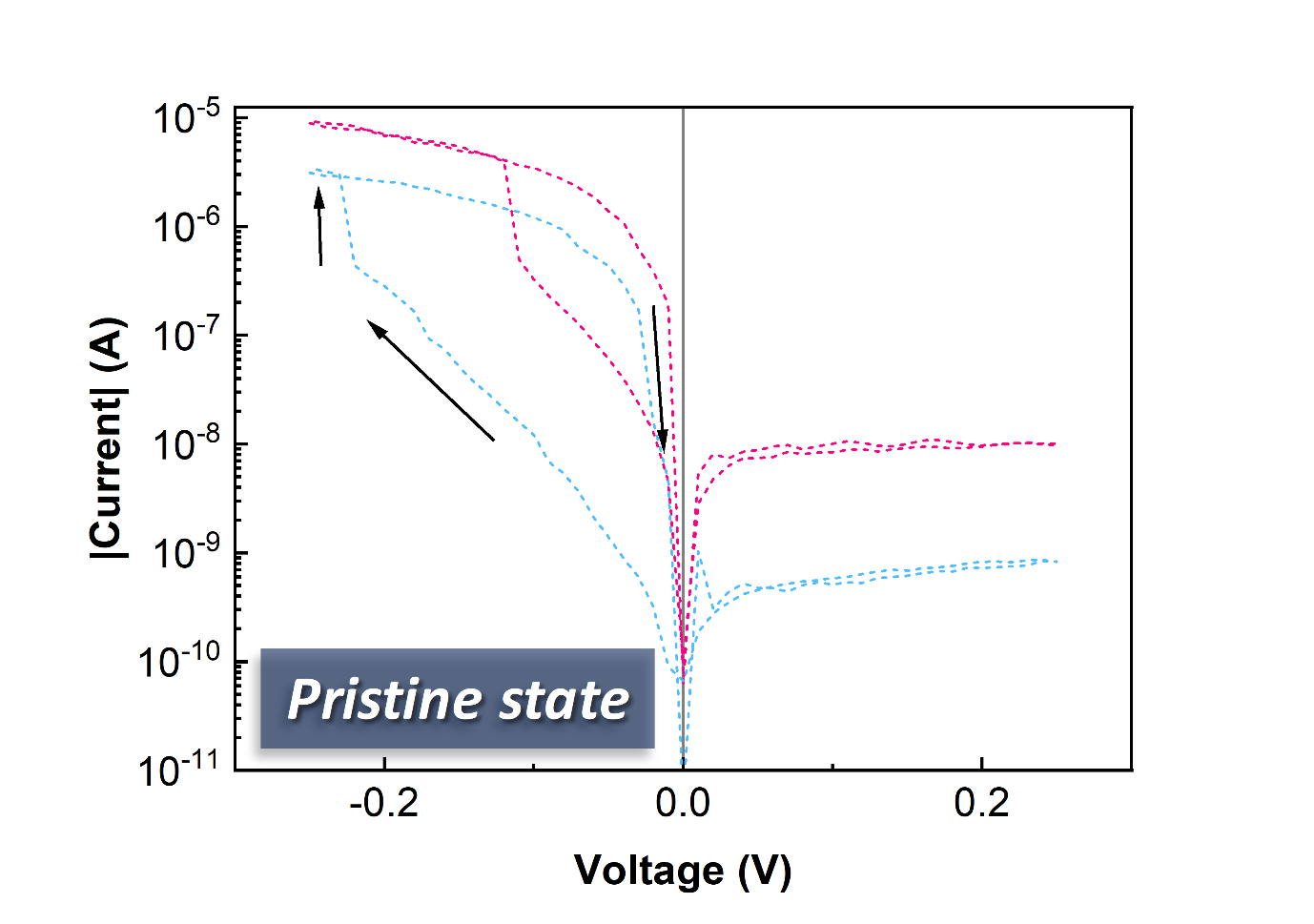


**Figure S6** - Examples of pristine curves of printed Ag/IGZO/ITO device. The curves were taken using a voltage sweep from -0.25V to 0.25V with a voltage step of 0.01V.

**Figure S7** - Linear characteristics of non-volatile switching.


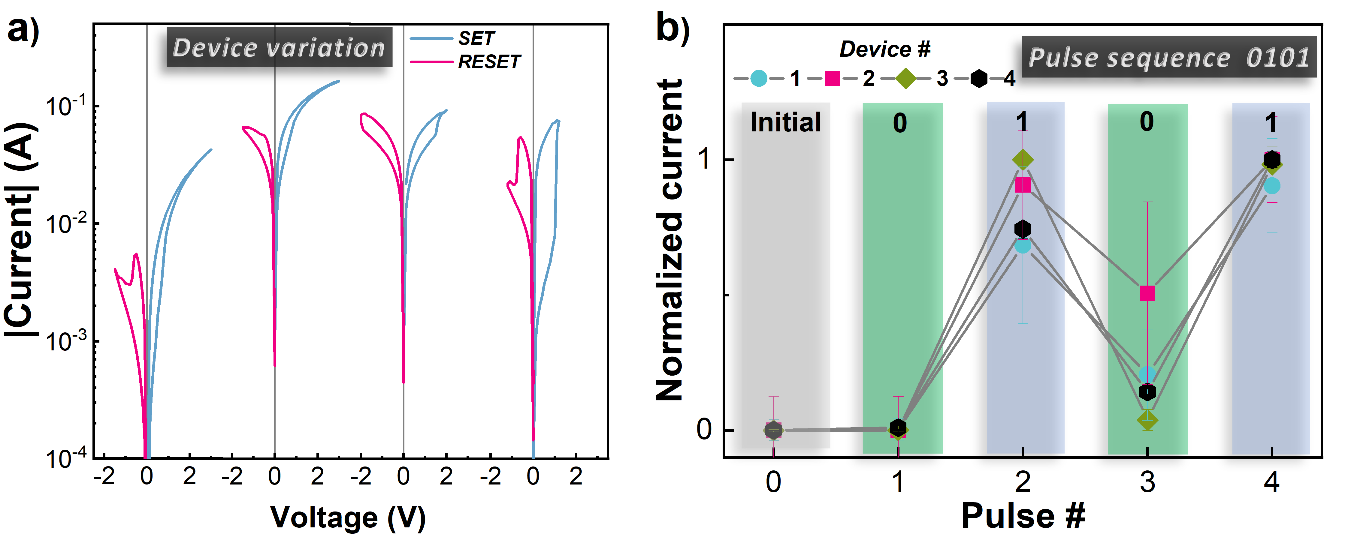


**Figure S8 -** Device variation: a) I-V characteristics (counter8wise switching - non-volatile) b) when applying the same pulse sequence (0101) and conditions: state 1 it was applied a -0.65 V pulse for 0.01 ms; the state 0 is 1 ms after the last “1” pulse; the reading was performed at 0.05 V for 1 ms.

**Table S1** - IGZO ink characteristics.

| **Parameter** | **Value** |
| --- | --- |
| Viscosity at 20 °C, 𝜂 (cP) | 4.16 |
| Surface tension at 20 °C, 𝛾 (mN/m) | 31.80 |
| Density, 𝜌 (g/cm^3^) | 0.966 |
| Drop diameter, d (µm) | 13 |
| Velocity, V (m/s) | 9.8 |
| Re | 30.0 |
| We | 38.4 |
| Oh | 0.21 |
| Z | 4.8 |

The Re, We, Oh and Z values were calculated using the equations below, where 𝜌 is the density of the fluid, V is its velocity, 𝜂 is its viscosity, 𝛾 is the surface tension, and d is a characteristic length: typically, the diameter of the jet, nozzle, or drop.

$$Z=\frac{1}{Oh} Oh=\frac{\sqrt{We}}{Re}=\frac{\eta}{\sqrt{\gamma\rho d}} Re=\frac{\rho Vd}{\eta};We=\frac{\rho V^{2}d}{\gamma}$$

*
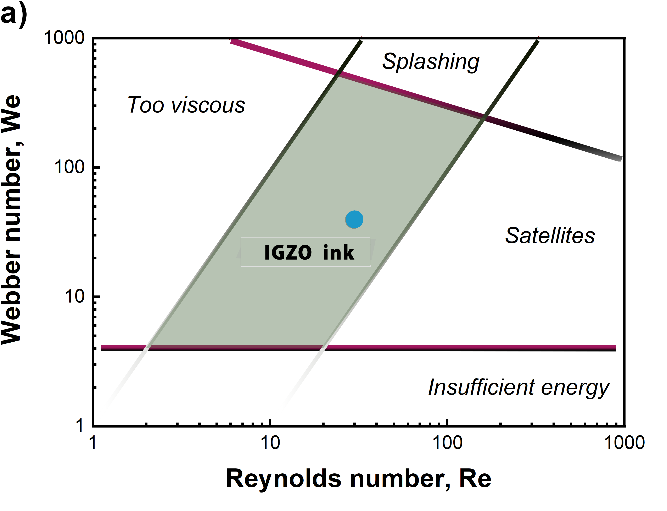

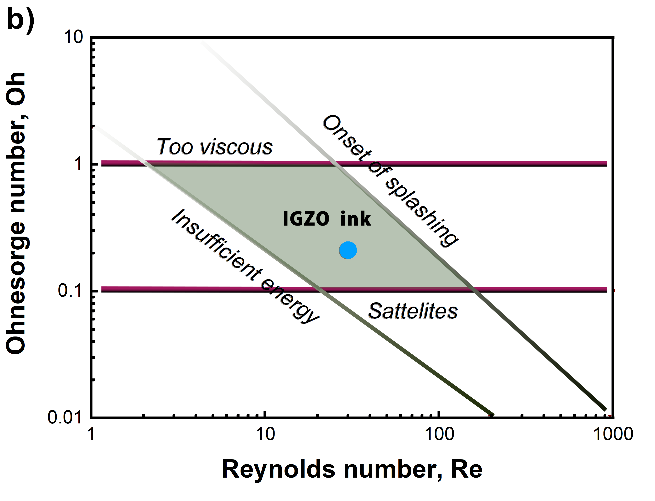
*

**Figure S9** - The operating regimes of drop formation in inkjet printing: (a) Weber number We versus Reynolds number Re, and (b) Ohnesorge number Oh versus Reynolds number Re. The developed IGZO ink is inside the optimal area for a stable drop formation.
